# Enhancing cardiology imaging: usability and implications of aortic annulus sizing software in transcatheter aortic valve replacement planning

**DOI:** 10.1186/s44348-024-00016-3

**Published:** 2024-08-05

**Authors:** Jah Yeon Choi

**Affiliations:** Cardiovascular Center, Korea University Guro Hospital, Korea University College of Medicine, Seoul, Republic of Korea

In cardiovascular imaging, supporting imaging software is pivotal in enhancing the accuracy, efficiency, and overall quality of diagnostic and interventional procedures. These software applications serve as indispensable tools for radiologists, cardiologists, and other healthcare professionals in the field as they can enhance the visualization and analysis of complex cardiovascular structures in high detail, support quantitative assessment, and enable post-processing tasks like image filtering, noise removal, and contrast enhancement, all of which contribute to clearer images [1, 2].

In the current issue of the *Journal of Cardiovascular Imaging*, Spanke et al. [3] conducted a comparative study on the usability and accuracy of two different aortic annulus sizing software programs, namely “3mensio Structural Heart (PIE Medical Imaging)” and “Valve ASSIST 2 (GE Healthcare),” for patients undergoing transcatheter aortic valve replacement (TAVR). This study is a significant contribution to the field of cardiology imaging, focusing on the critical aspect of preprocedural sizing in TAVR, which directly impacts patient outcomes. Despite the limited number of participants (11 for beginners in each imaging software group and nine for experts in the true reference group), the study’s methodology involved a randomized controlled design with both program-inexperienced users and experts, providing a comprehensive evaluation. The study demonstrated that Valve ASSIST 2 outperforms Structural Heart in terms of usability, with higher scores, faster measurements, and fewer questions needed. The detailed reporting on the percentage of correct valve size selections for both programs provides valuable insights into the real-world implications of usability on clinical decision-making. The authors also emphasize the need for further research involving experienced TAVR specialists, which is a logical next step to understanding the broader implications of software usability in the field.

Various aspects can evaluate imaging supporting software, such as usability, accuracy, speed, and efficiency, as demonstrated in the current study using tools like the System Usability Scale (SUS), ISONORM 9241/110-S questionnaire, the number of questions needed for measurements, and time on task. However, there are limitations in considering the average of experts’ measurement as an absolute true value. This suggests the necessity for standardized criteria to evaluate the numerous imaging software that has already been developed and will continue to be developed in the future [4, 5].

The future of cardiovascular imaging software is likely to be closely linked with artificial intelligence (AI). Machine learning algorithms can aid in automated disease detection, image analysis, and risk stratification. AI may assist in flagging anomalies in real time, thus expediting diagnosis and treatment and making it particularly suitable for handling the ever-growing volume of medical images in healthcare systems [6, 7].

In summary, this research contributes valuable insights into the usability of aortic annulus sizing software programs for TAVR planning. The well-structured study design and clear presentation of results make it a noteworthy addition to the field of cardiology imaging and hold promise for improving the precision of TAVR procedures.

## Data Availability

Not applicable.

## Abbreviations

AI: Artificial intelligence
TAVR: Transcatheter aortic valve replacement
